# Ontogeny of a tessellated surface: Carapace growth of the longhorn cowfish *Lactoria cornuta*

**DOI:** 10.1111/joa.13692

**Published:** 2022-05-31

**Authors:** Lennart Eigen, Daniel Baum, Mason N. Dean, Daniel Werner, Jan Wölfer, John A. Nyakatura

**Affiliations:** ^1^ Comparative Zoology Institute of Biology, Humboldt University of Berlin Berlin Germany; ^2^ Bernstein Center for Computational Neuroscience Berlin Humboldt University of Berlin Berlin Germany; ^3^ Visual and Data‐Centric Computing Department Zuse Institute Berlin Berlin Germany; ^4^ Department of Biomaterials Max Planck Institute of Colloids and Interfaces Potsdam Germany; ^5^ Department of Infectious Disease City University of Hong Kong and Public Health Kowloon Tong Hong Kong

**Keywords:** microCT, natural armor, ontogeny, *Ostraciidae*, tessellation

## Abstract

Biological armors derive their mechanical integrity in part from their geometric architectures, often involving tessellations: individual structural elements tiled together to form surface shells. The carapace of boxfish, for example, is composed of mineralized polygonal plates, called scutes, arranged in a complex geometric pattern and nearly completely encasing the body. In contrast to artificial armors, the boxfish exoskeleton grows with the fish; the relationship between the tessellation and the gross structure of the armor is therefore critical to sustained protection throughout growth. To clarify whether or how the boxfish tessellation is maintained or altered with age, we quantify architectural aspects of the tessellated carapace of the longhorn cowfish *Lactoria cornuta* through ontogeny (across nearly an order of magnitude in standard length) and in a high‐throughput fashion, using high‐resolution microCT data and segmentation algorithms to characterize the hundreds of scutes that cover each individual. We show that carapace growth is canalized with little variability across individuals: rather than continually adding scutes to enlarge the carapace surface, the number of scutes is surprisingly constant, with scutes increasing in volume, thickness, and especially width with age. As cowfish and their scutes grow, scutes become comparatively thinner, with the scutes at the edges (weak points in a boxy architecture) being some of the thickest and most reinforced in younger animals and thinning most slowly across ontogeny. In contrast, smaller scutes with more variable curvature were found in the limited areas of more complex topology (e.g., around fin insertions, mouth, and anus). Measurements of Gaussian and mean curvature illustrate that cowfish are essentially tessellated boxes throughout life: predominantly zero curvature surfaces comprised of mostly flat scutes, and with scutes with sharp bends used sparingly to form box edges. Since growth of a curved, tiled surface with a fixed number of tiles would require tile restructuring to accommodate the surface's changing radius of curvature, our results therefore illustrate a previously unappreciated advantage of the odd boxfish morphology: by having predominantly flat surfaces, it is the box‐like body form that in fact permits a relatively straightforward growth system of this tessellated architecture (i.e., where material is added to scute edges). Our characterization of the ontogeny and maintenance of the carapace tessellation provides insights into the potentially conflicting mechanical, geometric, and developmental constraints of this species but also perspectives into natural strategies for constructing mutable tiled architectures.

## Abstract 2

The carapace of boxfish is composed of mineralized polygonal plates, called scutes, arranged in a complex geometric pattern and nearly completely encasing the body. To clarify whether or how this armor is maintained or altered with age, we quantify architectural aspects of the carapace of the longhorn cowfish *Lactoria cornuta* through ontogeny, using high‐resolution microCT data and segmentation algorithms to characterize the hundreds of scutes that cover each individual.

## Graphical abstract

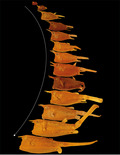

## 1 INTRODUCTION

Nature continues to provide inspiration for the design of innovative materials (Dunlop & Fratzl, 2010). In comparison with engineered materials, the elemental building blocks of biological materials are extremely limited and are combined into only two basic classes: biominerals (e.g., hydroxyapatite, calcium carbonate, and silica) and biopolymers (e.g., collagen, keratin, and chitin; Naleway et al., 2016). Due to this limited palette of building materials, evolution has arrived repeatedly on structural solutions that increase strength, toughness, flexibility, and fracture resistance (Dunlop & Fratzl, 2010; Fratzl et al., 2016). These offer ready fodder for bioinspired design in that their architectures often involve hierarchical arrangements of repeating geometric components, facilitating manufacture, and performance testing of biomimicked (e.g., 3D‐printed) models (e.g., Barthelat, 2007; Bonderer et al., 2008; Djumas et al., 2016; Domel et al., 2018; Mayer, 2005; Studart, 2012; Weaver et al., 2012).

Tessellations are common natural geometric architectures, involving arrays of hard geometric elements (tiles), often linked by softer connecting tissues (Fratzl et al., 2016). The diverse tessellations of vertebrate animals typically comprise mineralized (carbonated apatite‐based) tiles with collagenous links, as in the armored cartilage of sharks and rays (Dean et al., 2009; Seidel et al., 2016), armadillo osteoderms (Chen et al., 2011), turtle shells (Chen et al., 2015; Magwene & Socha, 2013), and the armors of many fishes (Kolmann et al., 2020; Woodruff et al., 2022; Yang et al., 2015). Tessellations can also provide fundamental structuring at far finer scale, as with mineralization foci (tesselles, ~2 μm long) in developing bone, which pack together in multilayered 3D formations to structure skeletal tissue (McKee et al., 2022). Biological tessellations can serve diverse roles (e.g., hydrodynamic or optical functions; Bartol et al., 2003; Domel et al., 2018; Li, Connors, et al., 2015a; Li, Kolle, et al., 2005), but larger‐scale armors most often play protective roles by minimizing transfer of mechanical stresses (e.g., from predation) to underlying soft tissues and vital organs (e.g., Bruet et al., 2008; Chen et al., 2011; Song et al., 2011; Taylor & Patek, 2010; Yang, Gludovatz, et al. 2013a; Yang, Chen, et al., 2013b). The architectural combination of soft and hard materials in tessellated armors can increase structural toughness (the energy absorbed before failure), with the many material interfaces controlling the initiation and propagation of cracks to localize damage (Dunlop & Fratzl, 2010; Dyskin et al., 2001; Fratzl et al., 2016; Mayer, 2005; Naleway et al., 2016). This containment of injury is vital, as failure of natural armors can create vulnerabilities to subsequent fights with conspecifics, predatory attacks, and/or infections (e.g., McClanahan & Muthiga, 1989; Reimchen, 1988; Zuschin et al., 2003). This highlights, however, an interesting conundrum in biological tessellations, in that they must maintain their structural integrity, even as the organism and the armor covering it grows.

In this study, we investigate the anatomy of the longhorn cowfish *Lactoria cornuta* (Linnaeus, 1758), a species characterized by a box‐like body shape and tessellated armor that encloses the body in a nearly complete shell (Yang et al., 2015). This armor is often referred to as a carapace (e.g., Bartol et al. 2002; Bartol et al., 2003; Yang et al., 2015). Boxfishes (Ostracioidea, within Tetraodontiformes) are found in the Atlantic, Pacific, and Indian Oceans, in warm tropical and subtropical waters, where they typically inhabit coral reefs and grass beds (Santini et al., 2013).

**FIGURE 1:**
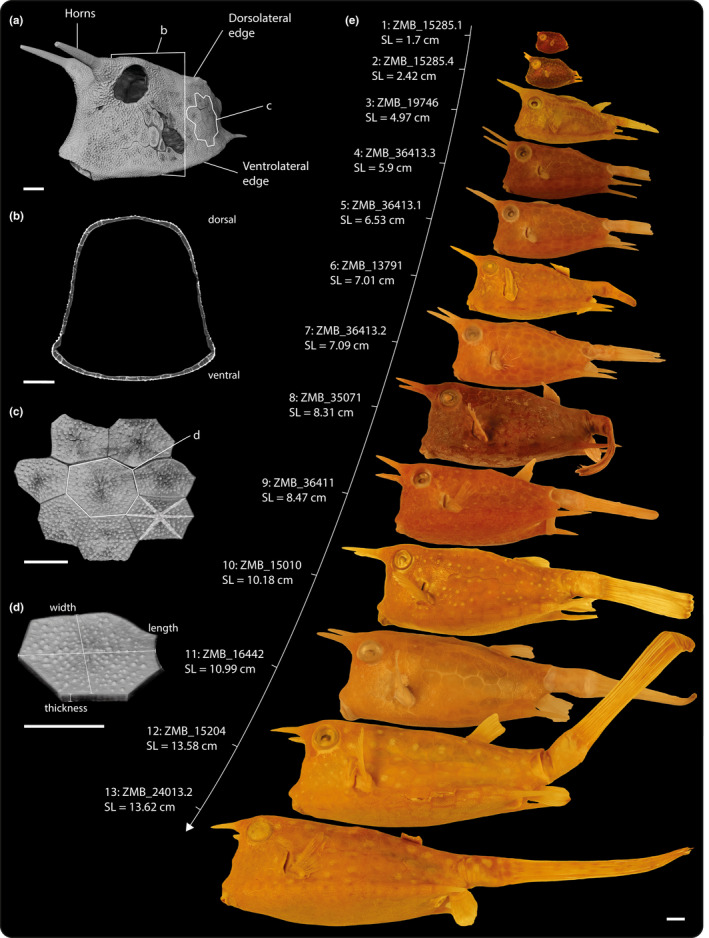
Specimens analyzed in this study. (a) Volume rendering of specimen ZMB_16442 with anatomical features listed. (b) Cross‐section of its carapace right behind its pectoral fins. (c) Closeup of scutes from its lateral side, with raised radial struts highlighted. (d) Single scute with principal dimensions. (e) Specimens of the longhorn cowfish *Lactoria cornuta* analyzed in this study, arranged by increasing standard length from top to bottom. Scale bars: 1 cm.

The boxfish carapace is a tessellation of scutes, geometric panels composed of a thin external mineralized plate bearing raised struts in a central star like pattern (highlighted in Figure 1c), sitting atop a thick base of layered, lightly‐mineralized collagen (Besseau & Bouligand, 1998; Santini et al., 2013; Tyler, 1980; Yang et al., 2015). Among the tens of thousands of bony and cartilaginous fish species, external body armors, and scalations are common, but structurally diverse. However, except in some heavily‐armored species with interlocking armors (e.g., bichir, gar, seahorses; Bruet et al., 2008; Porter et al., 2013; Yang, Gludovatz, et al., 2013a), fish scales, and scutes tend to be embedded in the skin quite separate from one another, with varying degrees of overlap (as in most fishes; Meyer & Seegers, 2012; Kolmann et al., 2020; Wainwright et al., 2018) or none at all (e.g., as in lumpsuckers; Woodruff et al., 2022). Whereas such overlapping or gapped armors allow a combination of flexibility and protection (e.g., Bruet et al., 2008; Lin et al., 2011; Vernerey & Barthelat, 2014; Yang, Gludovatz, et al., 2013a), as well as ready room for interstitial growth, the mineralized scutes of boxfish abut at their edges via jagged sutures, forming a relatively rigid encasement (Besseau & Bouligand, 1998; Naleway et al. 2016; Yang, Chen, et al., 2013b; Yang et al., 2015; Zhu et al., 2012). The interlocking and tight packing of the scutes therefore begs the question of how the tessellation accommodates body growth, especially since *L. cornuta* can attain maximum sizes up to ~40 cm standard length. It is, however, unknown how the carapace and its tessellation changes with age, and how the protective geometric patterning is maintained throughout the growth of the organism.

## 2 MATERIALS AND METHODS

### 2.1 Specimens

Thirteen specimens preserved in alcohol were obtained from the fish collection of the Museum für Naturkunde zu Berlin (MfN). The individual specimens examined varied by the factor of 8.5x in size, ranging from a standard length (tip of the mouth to the caudal peduncle) of 1.7–13.62 cm (Figure 1). Standard length was measured digitally in an extended version of the Amira software (AmiraZIBEdition 2020 and 2021, Zuse Institute).

### 2.2 MicroCT scanning

Samples were scanned at the Max Planck Institute of Colloids and Interfaces in Potsdam‐Golm (MPIKG) with an RX Solutions EASYTOM μCT scanner (RX Solutions). Scans for all samples were performed with helix scan sample rotation, at 100 kV source voltage, 150 μA source current, 15 W, and 333 ms exposure. A beam hardening correction algorithm was applied during image reconstruction.

### 2.3 CT image processing

**FIGURE 2:**
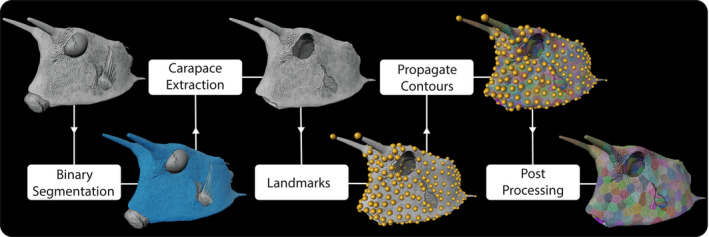
Semi‐automatic segmentation workflow of the carapace of the longhorn cowfish *Lactoria cornuta* from raw CT scan to individual tiles. The workflow was done in Amira. First, the carapace was segmented using the “Threshold” module. The portions that were not part of the carapace were removed manually. Landmarks were then placed manually onto each scute in a 3D surface visualization. The landmarks are the seeds for the “Propagate Contours” module, from which the subsequent segmentation propagates. See text for more information.

CT images were visualized and processed with AmiraZIBEdition. For segmentation of the carapace and individual scutes, since the close association of scutes did not allow a purely automatic threshold‐based segmentation, we adapted the semi‐automatic seed‐based segmentation workflow published by Baum et al. (2019). This approach involves manual placement of “seeds” in the individual structures to be segmented (e.g., the scutes), providing starting points from which the segmentation can grow (Figure 2). To briefly summarize the workflow, we first segmented the carapace based on its intensity values using the “Threshold” module. We then manually removed the portions that were not part of the carapace, such as the internal appendicular/axial skeleton, eyes, fins, and gills. Landmarks were then placed manually onto each scute in a 3D surface visualization of the carapace, created using the “Isosurface” module. The landmarks are the seeds for the “Propagate Contours” module, from which the subsequent segmentation propagates (i.e., one region per landmark). In the original version of the “Propagate Contours” module, voxels are added to a region according to decreasing intensity values. Here, we used a modified version, where the intensity values are divided by the 3D Euclidean distance of the voxel to the landmarks, thus, incorporating both intensity and distance information in the segmentation. The voxels are now traversed according to those modified intensity values, again in decreasing order. We used the modified version here because sometimes there was no clear valley in the intensity values between neighbored scutes. This resulted in greatly improved segmentations, requiring less of the postprocessing (manual refinement of segmentations) we found necessary for nearly all automated segmentations we tried. Undersegmented scutes (i.e., labels including >1 scute) were separated using the “Split Labels by Separation Surface” module, and oversegmented scutes (i.e., scutes divided into multiple labels) were merged using the “Pick and Merge Labels” module. This approach sometimes still overlooked errors. These remaining errors could be easily identified by looking at the region adjacency graph (RAG), which represents each label by a node, connected to its neighbors by linking edges (linear segments). The RAG was computed using the “Create Region Adjacency Graph” module. Manual merging of erroneous RAG nodes into other nodes was performed using the “Edit Spatial Graph with Line Raycast” module, which also allowed merging of the respective labels in the segmentations.

From the region adjacency graph (RAG), we then generated a surface using the “Create Surface Field from Spatial Graph Attribute” module, producing a clean representation of the segmented tessellation.

### 2.4 Surface area extraction

The carapace surface area (SA) of each boxfish was computed in AmiraZIBEdition from the generated surface with the “Surface Area Volume” module.

### 2.5 Carapace dimensions

If not specified otherwise, we used the software R version 4.1.2 (R Core Team, 2021) for all further analyses (see File S1 for R script) and the packages "readxl" (Wickham & Bryan, 2019), "tidyverse" (Wickham et al., 2019), and "egg" (Auguie, 2019) for data wrangling and data visualization. We removed the horns of each specimen as these were not of interest for this study. We used the scute coordinates to measure the length, height, and width of the carapace. The “prcomp” function was used to apply a principal component analysis to the *x*, *y*, and *z* coordinates of each boxfish separately. The first principal component always represented the anteroposterior axis, the second one the dorsoventral axis, and the third one the laterolateral axis. The absolute distance between the minimum and maximum values along each principal component was used to represent length, height, and width, respectively.

The three dimensions were natural log‐transformed and regressed on natural log‐transformed surface area using the “lm” function. The regression slope (i.e., scaling exponent) was reported and the “confint” function used to calculate its 95% confidence interval.

### 2.6 Number of scutes

The number of scutes was regressed on surface area using a generalized linear model with a Poisson distribution and a log‐link function as implemented in the “glm” function. The “confint” function was used to calculate the 95% confidence interval of the regression slope.

### 2.7 Scute analysis and visualization

The “Create Tesserae Statistics” module in AmiraZIBEdition was used to extract and characterize a variety of variables describing the morphology of scutes and their local environment. Input to the module were the label field containing the segmentation of the scutes, the RAG derived from this label field, and a surface representing the carapace shape. The following variables were computed: Firstly, (1) *number of neighbors*: number of neighboring scutes, evaluated on the RAG, as described above. Secondly, we quantified various scute dimensions, such as (2) *scute volume*: number of voxels × volume of a voxel; (3) *scute plane‐based area (PBA)*: the projected area of a scute, calculated for each scute by placing nodes in the centers of triangles/quads that surround the scute's vertex in the RAG. If the scute vertex was not completely surrounded by triangles or quads (e.g., it was bordering an opening in the carapace), then all plane‐based measures were set to −1000 to remove that scute from this variable's analysis. The least‐squares plane for the triangle/quad center nodes was calculated and that plane translated to contain the scute center, then the triangle/quad center nodes were projected onto the plane. PBA was calculated as the area of the polygon created by the projected nodes; (4) *scute thickness*: shortest dimension of a cuboid enclosing the scute. This measure captures the principal dimension of the scute orthogonal to the carapace surface; (5) *scute width*: largest principal dimension of the cuboid, perpendicular to the thickness dimension (corresponds to scute length in Figure 1d). These morphological descriptors are intended to characterize scute gross morphology but don't capture differences in ultrastructural features (e.g., interscute differences in their raised radial struts). Finally, we also analyzed the local carapace curvature around each scute using (6) *Gaussian surface curvature*: the product of the two principal curvature values of the surface field at the location of the scute's center; (7) *mean surface curvature*: the mean of the two principal curvature values of the surface field at the location of the scute's center. The relative frequency of the number of neighbors was plotted for each specimen, which were ordered by increasing size to assess scaling effects. Here and below, we always used SA as a size indicator. All raw data can be found in Table S1.

The scaling of the scute dimensions with boxfish size was analyzed by linearly regressing the median of the scute dimensions on SA across the 13 specimens. The variables were natural log‐transformed prior to analysis and the same R functions as explained for the carapace dimensions were used. For the purpose of investigating the variability of scute dimensions within boxfishes, we also performed boxplots of normalized dimension values ordered by increasing specimen size. Volume was normalized by dividing with SA^3/2^, PBA by dividing with SA, and thickness and width by dividing with SA^1/2^, respectively.

The trend of the median and the variability of the curvature variables were also analyzed by normalization and the generation of boxplots ordered by specimen size. For this purpose, Gaussian surface curvature was multiplied with SA and mean surface curvature multiplied with SA^1/2^.

The variables above capture scute properties, but with no positional determinants or relationship to anatomical location. To compare different body regions (i.e., to look for the association between scute properties and specific anatomical location), four specimens of different sizes were chosen as examples, and their scutes were color‐coded in AmiraZIBEdition according to all scute parameters.

## 3 RESULTS

### 3.1 Carapace shape but not scute number changes with boxfish size

**FIGURE 3:**
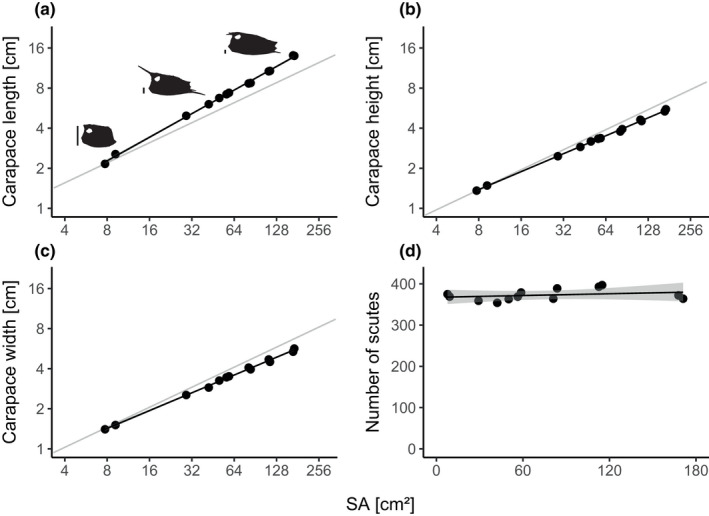
Regression plots of carapace dimensions and number of scutes versus carapace surface area (SA) in the longhorn cowfish *Lactoria cornuta*. (a–c) Carapace dimensions. The gray line indicates isometric scaling. Shaded areas indicate 95% confidence interval of the model parameters from a linear regression model, but are barely visible due to the strong linearity of the relationships. Log–log‐scales were used. Three exemplary boxfish silhouettes (ZMB_15285.1, ZMB_36413.2, ZMB_24013.2) in (a) were scaled to equal carapace height (excluding horns) to illustrate relative elongation of the carapace with increasing body size. Scale bars: 1 cm. Relative elongation becomes evident due to carapace length scaling with positive allometry and carapace height with negative allometry relative to SA. (d) Number of scutes. Shaded area indicates the back‐transformed (exponentiated) 95% confidence interval of the model parameters from a Poisson regression model with a log‐link function.

Boxfish carapace length scaled with positive allometry with regard to SA (Figure 3). The slope and its confidence interval were 0.59 and [0.57; 0.60], respectively, where as a slope of 0.5 would indicate isometric scaling. Carapace height and width both scaled with negative allometry with regard to SA (Figure 3). The slope and its confidence interval were 0.45 and [0.44; 0.46], respectively, for height, and 0.44 and [0.43; 0.46], respectively, for width, whereas a slope of 0.5 would indicate isometric scaling. These scaling relationships of length and height reflect the changing carapace shape with size in the form of relative anteroposterior elongation (Figure 1a).

Scute number did not vary significantly with SA (regression slope: 1.0002; confidence interval: [0.9996; 1.0007]; Figure 3). For example, the individuals with the smallest (1.7 cm) and the largest (13.6 cm) SLs in our dataset had 375 and 364 scutes, respectively. Overall, the minimum and maximum observed numbers of scutes were 354 (4.2 cm SL) and 397 (101.8 cm SL), respectively, indicating no substantial change in scute number with SA.

### 3.2 Number of neighbors does not change with boxfish size

**FIGURE 4:**
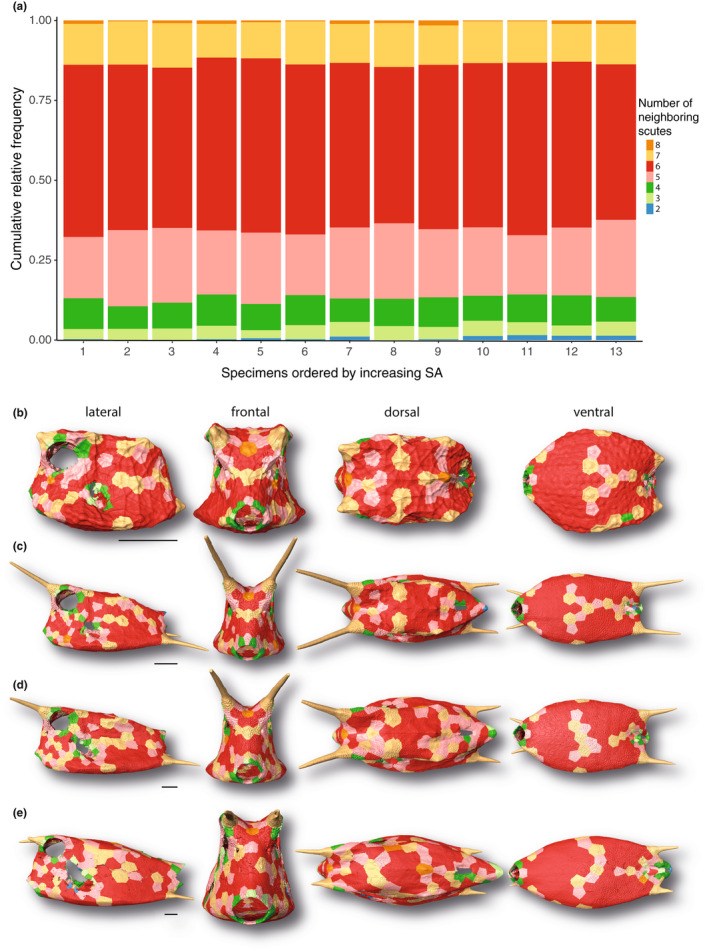
Relative frequency and regional differentiation of the number of scute neighbors in the longhorn cowfish *Lactoria cornuta*. (a) Cumulative relative frequency of the number of scute neighbors within each studied specimen Specimens are ordered by increasing carapace surface area. (b–e) Surface renderings of carapaces from four exemplary boxfishes with different standard lengths and scutes color‐coded according to their number of neighboring scutes. The smallest specimen (b 1.7 cm), two medium‐sized specimens (c 6.53 cm; d 8.47 cm), and the largest specimen (e 13.62 cm) are shown (see Figure 1). Horns are color‐coded but were not analyzed in this study. Image columns from left to right: left lateral, frontal, dorsal, and ventral views. Scale bars: 1 cm.

The carapace was mostly composed of hexagonal scutes (i.e., with six neighboring scutes) (~49% to 55% of all scutes), followed by scutes with five (~19% to 24%) and seven neighbors (~11% to 14%). Very few scutes had four (~7% to 10%), or three neighbors (~3% to 5%) (Figure 4a). Scutes with one, two, or eight neighbors were almost non‐existent (~0% to 2%), and only one individual boxfish had a scute with nine neighbors. Openings of the carapace (e.g., for the mouth and eyes) were often surrounded by scutes with just four or five neighbors (Figure 4b‐e). Otherwise, the number of neighbors did not appear to be linked to anatomical region. Only the posterior ventrolateral and ventral sides were more consistently covered by hexagonal scutes compared to other regions of the carapace. The frontal, dorsal, and ventral views in Figure 4 suggest a certain degree of bilateral symmetry regarding the number of neighbors.

**FIGURE 5:**
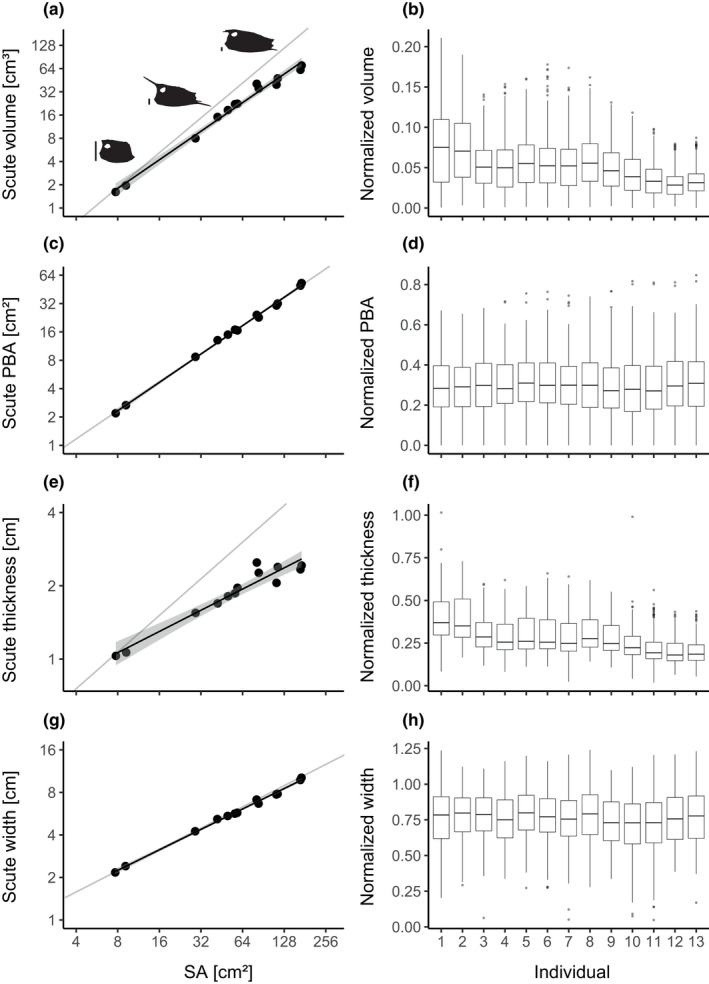
Regression plots and boxplots of scute dimensions versus carapace surface area (SA) for an ontogenetic series of the longhorn cowfish *Lactoria cornuta*. (a), (c), (e) and (g) Regression plots of the median values of the scute dimensions versus SA. Log–log‐scales were used. The gray line indicates isometric scaling. Shaded areas indicate 95% confidence interval of the model parameters from a linear regression model but are sometimes barely visible due to the strong linearity of the relationships. Three exemplary boxfish silhouettes (ZMB_15285.1, ZMB_36413.2, ZMB_24013.2) in (a) were scaled to equal carapace height (excluding horns) to illustrate relative lengthening of the carapace with increasing body size. Scale bars: 1 cm. (b), (d), (f) and (h) Boxplots of normalized scute dimensions ordered by increasing SA. PBA, plane‐based area.

### 3.3 Scute morphometrics vary in their scaling properties

Scute volume scaled with negative allometry (i.e., with a slope of 1.23; Figure 5a). The isometric scaling relationship of 1.5 was not included in the 95% confidence interval ([1.14; 1.32]). The within‐individual variability of the normalized volume slightly decreased with increasing size of the boxfish (i.e., scutes become more uniform in their normalized volume; Figure 5b).

Scute PBA scaled isometrically with a slope of 1 and a confidence interval of [0.97; 1.03] (Figure 5c). The variability of the PBA appeared to remain constant with increasing size according to Figure 5d.

Scute thickness scaled also with negative allometry (0.29, the isometric scaling relationship of 0.5 was not included in the 95% confidence interval [0.24; 0.34]; Figure 5e). Similar to normalized volume, the variability of the normalized thickness decreased slightly with size (Figure 5f).

Scute width scaled with negative allometry (0.48), but isometric scaling (i.e., a slope of 0.5) could not be excluded as the true relationship since it was included in the 95% confidence interval ([0.46; 0.50]; Figure 5g). Similar to normalized PBA and in contrast to normalized volume and thickness, the variability of the normalized width of scutes remained constant with size (Figure 5h). For a given SA, median scute width was always larger than median scute thickness, illustrating the plate‐like morphology of most scutes (Figure 5f, h).

### 3.4 Scute morphometrics vary among carapace regions

**FIGURE 6:**
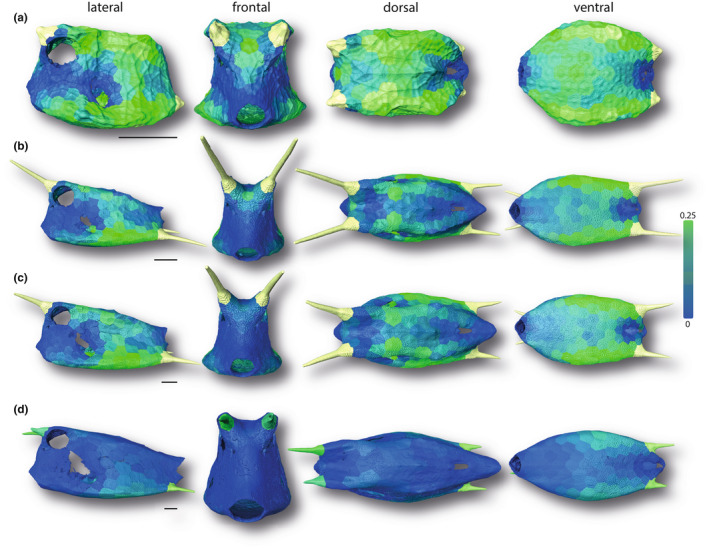
Surface renderings of boxfish carapaces with scutes color‐coded according to their normalized volume. Green and blue indicate regions with larger and smaller normalized volumes, respectively. The smallest specimen (a 1.7 cm), two medium‐sized specimens (b 6.53 cm; c 8.47 cm), and the largest specimen (d 13.62 cm) are shown (see Figure 1). Horns are color‐coded but were not analyzed in this study. Image columns from left to right: left lateral, frontal, dorsal, and ventral views. Scale bars: 1 cm.

The normalized scute dimensions varied among different regions of the carapace of an individual but also within the same region among individuals of different sizes. With regard to volume, the head, anus, and fin insertions were generally covered by relatively less massive scutes (Figure 6). In contrast, the ventral side of all specimens was covered by scutes with a larger volume compared to the lateral, frontal, and caudal sides. Also, scutes at the dorsolateral and ventrolateral edges had a larger volume in all specimens, compared to scutes at the lateral, dorsal, and ventral sides. The negative allometric scaling relationship of absolute scute volume with SA (Figure 5) was reflected by scutes of all body regions equally displaying a subsequent decrease of normalized volume with body size (Figure 6).

**FIGURE 7:**
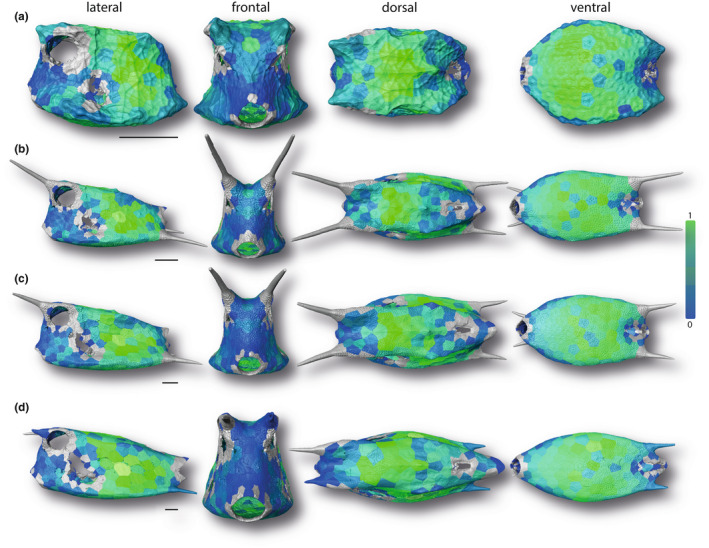
Surface renderings of boxfish carapaces with scutes color‐coded according to their normalized plane‐based area (PBA). Green and blue indicate regions with larger and smaller normalized PBAs, respectively. Gray indicates regions with scutes whose PBA could not be computed. The smallest specimen (a 1.7 cm), two medium‐sized specimens (b 6.53 cm; c 8.47 cm), and the largest specimen (d 13.62 cm) are shown (see Figure 1). Horns are color‐coded but were not analyzed in this study. Image columns from left to right: left lateral, frontal, dorsal, and ventral views. Scale bars: 1 cm.

As stated above, the method used for calculating PBA precluded determining the normalized PBA for most of the scutes around the carapace openings (Figure 7). Similar to volume, the head, anus, and fin insertions are generally covered by relatively less massive scutes in terms of PBA (Figure 7). In contrast, the ventral, lateral, and dorsal sides as well as the dorsolateral and ventrolateral edges of all specimens were covered by scutes with a relatively larger PBA. The isometric scaling relationship of absolute scute PBA with SA (Figure 5) is reflected by scutes of all body regions remaining fairly constant in terms of normalized PBA across body sizes (Figure 7).

**FIGURE 8:**
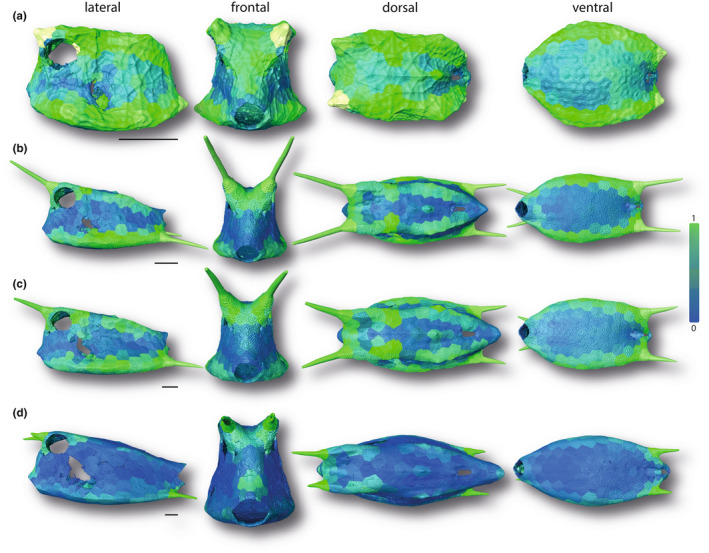
Surface renderings of boxfish carapaces with scutes color‐coded according to their normalized thickness. Green and blue indicate regions with larger and smaller normalized thickness, respectively. The smallest specimen (a 1.7 cm), two medium‐sized specimens (b 6.53 cm; c 8.47 cm), and the largest specimen (d 13.62 cm) are shown (see Figure 1). Horns are color‐coded but were not analyzed in this study. Image columns from left to right: left lateral, frontal, dorsal, and ventral views. Scale bars: 1 cm.

With regard to thickness, the scutes of the anterior head region, fin insertions, as well as of the lateral, dorsal, and ventral sides appeared to be relatively less massive compared to the scutes around the anus and those of the dorsolateral and ventrolateral edges (Figure 8). The negative allometric scaling relationship of absolute scute thickness with SA (Figure 5) was reflected by scutes of all body regions equally displaying a subsequent decrease of normalized thickness with body size (Figure 8).

**FIGURE 9:**
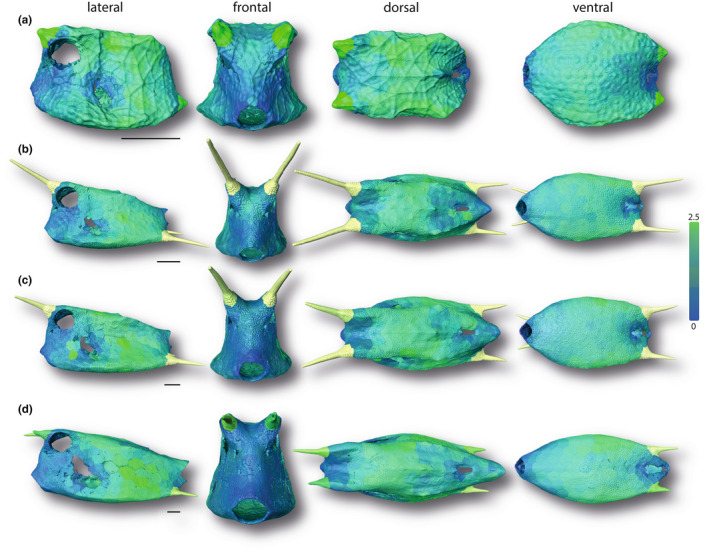
Surface renderings of boxfish carapaces with scutes color‐coded according to their normalized width. Green and blue indicate regions with larger and smaller normalized width, respectively. The smallest specimen (a 1.7 cm), two medium‐sized specimens (b 6.53 cm; c 8.47 cm), and the largest specimen (d 13.62 cm) are shown (see Figure 1). Horns are color‐coded but were not analyzed in this study. Image columns from left to right: left lateral, frontal, dorsal, and ventral views. Scale bars: 1 cm.

The results of the normalized scute width mirrored the results of the normalized PBA. The head, anus, and fin insertions were generally covered by relatively less massive scutes in terms of width (Figure 9). Also, in contrast, the ventral, lateral, and dorsal sides as well as the dorsolateral and ventrolateral edges of all specimens were covered by scutes with a relatively larger width. As for PBA, the isometric scaling relationship of absolute scute width with SA (Figure 5) was reflected by scutes of all body regions remaining fairly constant in terms of normalized width across body sizes (Figure 9).

### 3.5 Local surface curvature varies among carapace regions

We used two metrics for quantifying carapace surface curvature local to individual scutes. The Gaussian curvature (the product of the two local principal curvatures) indicates whether a surface is saddle‐like (negative), or sphere‐like (positive). If the Gaussian curvature is zero either the surface is flat at this point (i.e., both principal curvatures are zero) or only one principal curvature is zero, as in a long ridge or valley. These two cases of zero Gaussian curvature can only be distinguished by looking at a second metric, the mean curvature (the average of the two local principal curvatures), which represents the relationship between convexity and concavity of the principal curvatures of a surface. Sphere‐like surface regions possess large absolute values, whereas all other regions, flat and saddle‐shaped, tend toward zero. Therefore, in long ridges or valleys, where Gaussian curvature is zero, the mean curvature will be either positive for a convex curvature or negative for a concave curvature.

**FIGURE 10:**
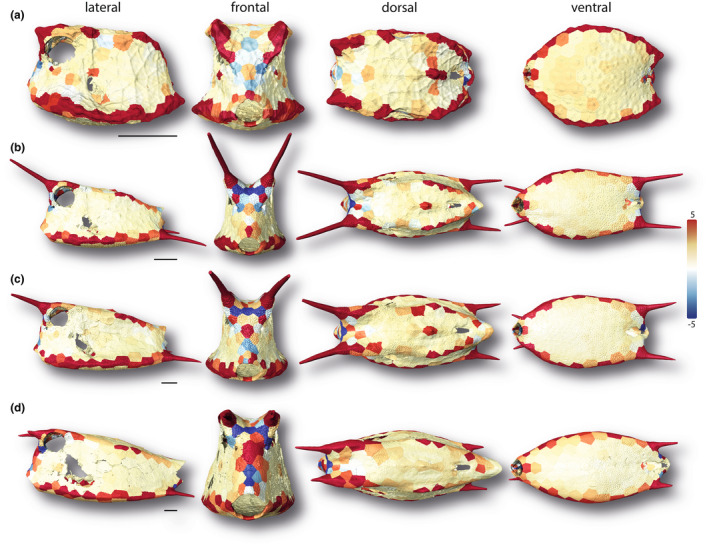
Surface renderings of boxfish carapaces with scutes color‐coded according to their normalized Gaussian curvature. Values above 5 are colored in red; Values below −5 are colored in blue. The smallest specimen (a 1.7 cm), two medium‐sized specimens (b 6.53 cm; c 8.47 cm), and the largest specimen (d 13.62 cm) are shown (see Figure 1). Horns are color‐coded but were not analyzed in this study. Image columns from left to right: left lateral, frontal, dorsal, and ventral views. Scale bars: 1 cm.

Our measures of normalized curvature allowed comparison of the general carapace shape across individuals, ignoring the effect of size. For example, in all specimens, on the lateral, ventral, and dorsal sides, the normalized Gaussian curvature of the scutes was positive but close to zero in all specimens (Figure 10). The regions around the anus and on the cranium between the eyes and between the cranial horns consisted of relatively more scutes with negative values (<−5), indicating a saddle‐shaped surface, whereas the dorsolateral and ventrolateral edges were made up of scutes with the highest positive values (>5), indicating raised spheroidal surfaces. The median normalized Gaussian curvature and its variability did not appear to change with increasing body size (Figure S1).

**FIGURE 11:**
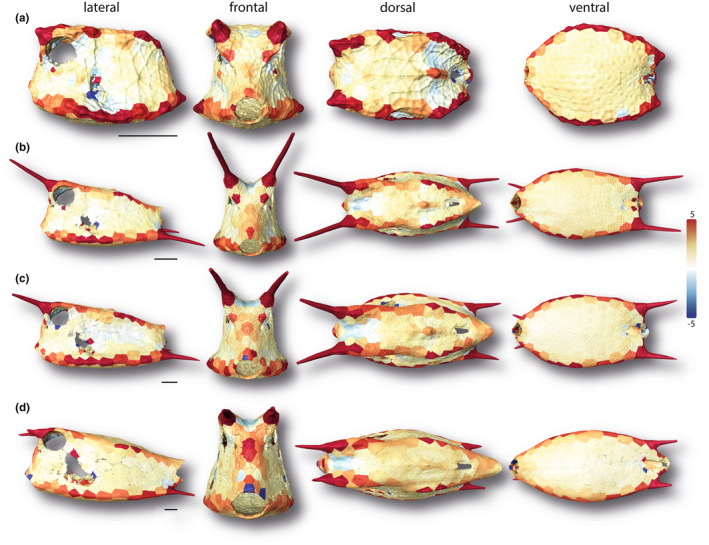
Surface renderings of boxfish carapaces with scutes color‐coded according to their normalized mean curvature. Values above 5 are colored in red; Values below −5 are colored in blue. The smallest specimen (a 1.7 cm), two medium‐sized specimens (b 6.53 cm; c 8.47 cm), and the largest specimen (d 13.62 cm) are shown (see Figure 1). Horns are color‐coded but were not analyzed in this study. Image columns from left to right: left lateral, frontal, dorsal, and ventral views. Scale bars: 1 cm.

With regard to normalized mean curvature, individuals were fairly similar in being evenly covered by scutes with a relative mean curvature close to zero (depicted with pale orange color; Figure 11). The craniodorsal region between and behind the cranial horns consisted generally of more scutes with values close to zero, whereas the dorsolateral and ventrolateral edges were made up of scutes with the highest positive values. The median normalized mean curvature and its variability did also not appear to change with increasing body size (Figure S1). However, regional trends with body size could be observed at the lateral and the caudodorsal regions. In both areas, the mean curvature of the scutes increased from positive values close to zero (whitish color of scutes in Figure 11) to more positive values (yellowish color of scutes in Figure 11). Only a few single scutes around carapace openings were characterized by a negative mean curvature.

## 4 DISCUSSION

A growing armor is a structural design challenge, in that it must change to accommodate an ever‐larger animal, while maintaining its structural integrity for protection. A tessellated armor can be valuable in this regard, in that individual elements can conceivably be added, altered, or even replaced, with minimal disruption to the integrity of the protective covering (Estrin et al., 2021; Fratzl et al., 2016).

Our microCT and quantitative image analyses revealed a consistent growth pattern of the longhorn cowfish carapace, reflected in the narrow confidence intervals recovered in all parameter regressions on surface area. The carapace becomes larger with age but also changes its aspect ratio from near‐spherical to a rectangular cuboid morphology. This is accomplished by a constant relative elongation of the body along the anteroposterior axis and relative lateromedial narrowing, as indicated, respectively, by the strong positive allometric scaling of carapace length as well as negative allometric scaling of carapace height, and the negative allometric scaling of body width relative to surface area (Figure 3). This carapace growth is not accomplished by the addition of new scutes, as in the armor of the Pacific spiny lumpsucker, where smaller odontodes arise in gaps between larger ones (Woodruff et al., 2022). Rather, cowfish armors, like the keratinous scutes of turtle shells (Alibardi, 2005; Wyneken et al., 2007), establish a set number of scutes early in life (~370 on average) and those scutes are then enlarged during ontogeny. This explains previous anecdotal observations of different scute sizes in individuals of different ages (Yang et al., 2015).

Instead of individual scutes becoming relatively more massive with age, as with lumpsucker odontodes (Woodruff et al., 2022), the comparatively thicker‐walled scutes of younger cowfish become wider but relatively thinner in adults (although, in an absolute sense, more than 2× as thick as scutes of the youngest animals in our study). The relative thinning of scutes is comparable to the tessellated cartilage of stingrays, where the tesserae that form the tiled crust of the skeleton grow more rapidly in width than thickness, resulting in a comparative thinning of the skeletal cortex as animals age (Dean et al., 2009). From a gross mechanical standpoint, this is a relatively material‐efficient solution: the second moment of area of an object, an indicator of its resistance to bending, increases rapidly as material is arranged further from the shape's neutral bending axis (Frank, 2014; Habegger et al., 2015; Summers et al., 2004). In principle, from a purely geometric perspective, the relatively thinner carapace of large cowfish is therefore a mechanically efficient use of material, whereas smaller individuals (which have not yet attained body sizes that afford higher bending resistance) rely on thicker armor and body cross‐sections more reminiscent of architectural I‐beams (narrowed at the midbody, wider at the top and bottom; e.g., see frontal view in Figure 4a). The width‐thickness ratio of scales in the cowfish armor should also play a role in determining how much individual scutes bend in response to mechanical insult (e.g., predatorial attack; Browning et al., 2013; Vernerey & Barthelat, 2014), meaning the higher aspect ratio scutes of older animals may be riskier for certain types of damage (e.g., from puncture). The layered tissue architecture of scutes, the tooth‐like sutures linking them, and the raised stellate ridges on scute surfaces (Besseau & Bouligand, 1998; Yang et al., 2015) likely provide reinforcement to mitigate these effects. Mechanical property and mineral density variation of scutes, both within individuals and across different ontogenetic stages, will also affect the carapace's protective abilities and warrant future study. An examination of the mechanical interaction of carapace gross morphology with its tessellation pattern and scute microstructures (e.g., ridges and sutures) would clarify how multi‐scale architectural and material features trade‐off mechanically (e.g., in a balance of stiffness and toughness).

Our curvature data illustrate that the common moniker “boxfish” is well deserved. Like a box, the cowfish carapace is predominantly a zero (Gaussian and mean) curvature shape, with larger positive curvatures only along its edges. As observed by previous authors (Besseau & Bouligand, 1998; Yang et al., 2015), we found the carapace to be crafted predominantly by hexagonal scutes, balanced mostly by five‐ and seven‐sided scutes, similar to stingray tessellated cartilage and as expected for a closed, convex three‐dimensional surface (Baum et al., 2019). Our results show this distribution of tile shapes is independent of animal size (Figure 4). With the left–right symmetry of the scute pattern (Yang et al., 2015), and the static number of scutes we demonstrated for cowfish, this argues for an underlying developmental program that canalizes growth and reduces variability. The regulating factors involved in the initial development of scutes in young animals deserve investigation, as this critical timepoint apparently establishes scute distribution and number for life. The question of how growth is coordinated at scute margins is particularly intriguing, given that the sutural gap between the outer mineralized plates' of neighboring scutes is apparently oddly devoid of cells and tissues (Yang et al., 2015). The fibrous joints linking scute bases are therefore exciting regions to explore, since these are likely involved in both active mineralization (to grow scutes), but also local inhibition of mineralization (to maintain the flexible non‐mineralized joints between them, as proposed for tessellated cartilage; Seidel et al., 2016).

The box‐like morphology of the cowfish carapace is ideal for a tessellated growth system where the number of tiles is fixed: a cylindrical body cross‐section would require considerable structural reshaping of individual tiles in order to remodel the high tile curvatures of young animals for adult body sizes. In contrast, with a boxy carapace shape no remodeling is required, tiles can simply grow at their margins to increase body sizes. This is facilitated by the scutes of cowfish having limited “global” (whole‐scute) curvature, rather only local curvature: individual scutes are predominantly either relatively flat or exhibit localized bends to form carapace edges (Figures 10, 11). Corners and edges are weak points in boxy architectures (Frank, 2014), and edge scutes are comparatively thick in smaller cowfish (Figure 8), only eventually becoming comparatively thinner in larger carapaces with higher bending resistance. In the cowfish “assembly” system—predominantly flat “wall scutes” and bent “edge scutes”—individual tiles can attain comparatively large sizes relative to animal body size, since larger curvatures are limited and very localized to individual scutes (ventrolateral edges) or the joints between them (dorsolateral edges). This is in striking contrast to tessellated cartilage, where skeletons are more rounded in cross section and armored with thousands of tiny tiles in order to conform to high surface curvatures (Baum et al., 2019; Maisey et al., 2021). Indeed, besides at the carapace edges, the cowfish architectural system of predominantly flatter, larger tiles and lower surface curvatures only deviates around carapace openings and more finescale topologies (e.g., between the eyes), where smaller scutes shape more complicated curvatures.

Our findings demonstrate how the combined effects of body geometry and tessellation can provide a biological solution for the “problem” of growing a tiled surface. The boxy body morphology of the longhorn cowfish *L. cornuta* permits a canalized developmental system where an armor with a fixed number of scutes and limited scute shapes can develop through a comparatively simple accretionary growth strategy (i.e., without remodeling being necessary), over a large range of body size, while maintaining armor integrity. Future work will further clarify constructional constraints in the development and evolution of this tessellated armor across the diverse polygonal body cross sections of boxfishes (Ostracioidea; Bartol et al., 2005).

## Supporting information


Figure S1

Table S1

DataS 1

## Data Availability

The scan data support the findings of this study are available from the corresponding author LE upon request.
