# Supplementary material for: Ontogeny of a tessellated surface: Carapace growth of the longhorn cowfish Lactoria cornuta
Source: J Anat. 2022 May 31;241(3):565–80. doi: 10.1111/joa.13692 (PMC9358767; doi:10.1111/joa.13692)
Supplement: Supplementary file 1 — Figure S1 [file JOA-241-565-s003.pdf]

Supplementary figures for

Ontogeny of a tessellated surface: carapace growth of the longhorn cowfish *Lactoria cornuta*

Lennart Eigen*[1,2], Daniel Baum[3], Mason N. Dean[4,5], Daniel Werner[4], Jan Wölfer[1], John A. Nyakatura[1]

*corresponding author: lennart.eigen@hu-berlin.de

1: Humboldt University of Berlin, Institute of Biology, Comparative Zoology, Philippstraße 13, 10115 Berlin, Germany.

2: Bernstein Center for Computational Neuroscience Berlin, Humboldt University of Berlin, Philippstraße 13, 10115 Berlin, Germany

3: Zuse Institute Berlin, Visual and Data-Centric Computing Department, Takustraße 7, 14195 Berlin, Germany

4: Max Planck Institute of Colloids and Interfaces, Department of Biomaterials, Am Mühlenberg 1, 14424 Potsdam, Germany.

5: *Current address:* City University of Hong Kong, Department of Infectious Disease and Public Health, Kowloon Tong, Hong Kong

Keywords: Tessellation, *Ostraciidae,* Ontogeny, Natural armor, MicroCT

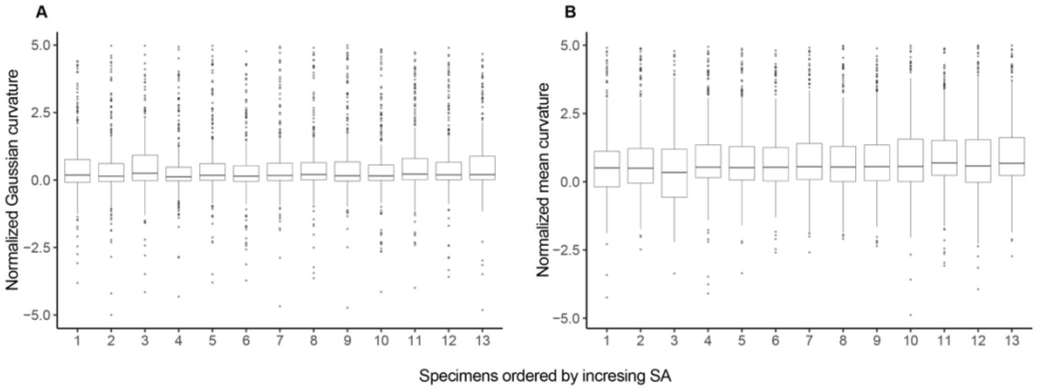

Figure S1 | Boxplots of normalized local curvature for an ontogenetic series of the longhorn cowfish *L. cornuta*. Boxplots are ordered by increasing carapace surface area (SA). **A** Normalized Gaussian curvature. **B** Normalized mean curvature. Extreme outliers (values < -5 or > 5) were removed to improve visibility of major trends.
